# Protein–protein binding pathways and calculations of rate constants using fully-continuous, explicit-solvent simulations†
†Electronic supplementary information (ESI) available. See DOI: 10.1039/c8sc04811h


**DOI:** 10.1039/c8sc04811h

**Published:** 2018-12-27

**Authors:** Ali S. Saglam, Lillian T. Chong

**Affiliations:** a University of Pittsburgh , Department of Chemistry , 219 Parkman Avenue , Pittsburgh , PA 15260 , USA . Email: ltchong@pitt.edu ; Tel: +1-412-624-6026

## Abstract

A grand challenge in the field of biophysics has been the complete characterization of protein–protein binding processes at atomic resolution. This characterization requires the direct simulation of binding pathways starting from the initial, unbound state and proceeding through states that are too transient to be captured by experiment. Here, we applied the weighted ensemble path sampling strategy to orchestrate atomistic simulation of protein–protein binding pathways. Our simulation generated 203 fully-continuous and independent pathways along with rate constants for the binding process involving the barnase and barstar proteins. Results reveal multiple binding pathways along a “funnel-like” free energy landscape in which the formation of the “encounter complex” intermediate is rate-limiting followed by a relatively rapid rearrangement of the encounter complex to the bound state. Among all diffusional collisions, only ∼11% were productive. In the most probable binding pathways, the proteins rotated to a large extent (likely *via* electrostatic steering) in order to collide productively followed by “rolling” of the proteins along each other's binding interfaces to reach the bound state. Consistent with experiment, R59 was identified as the most kinetically important barnase residue for the binding process. Furthermore, protein desolvation occurs late in the binding process during the rearrangement of the encounter complex to the bound state. Notably, the positions of crystallographic water molecules that bridge hydrogen bonds between barnase and barstar are occupied in the bound-state ensemble. Our simulation was completed in a month using 1600 CPU cores at a time, demonstrating that it is now practical to carry out atomistic simulations of protein–protein binding.

## Abstract 2

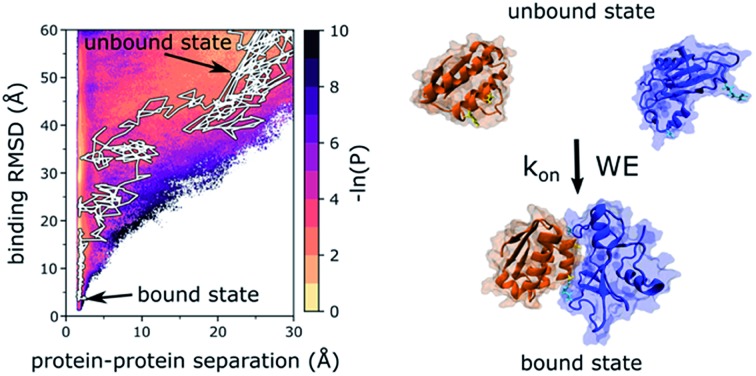
The weighted ensemble (WE) strategy enables direct simulation of atomistic, fully-continuous protein–protein binding pathways in explicit solvent, yielding rigorous kinetics.

## 1. Introduction

Life is enabled by protein–protein interactions. Each of these interactions involves an elaborate molecular dance of the partner proteins until they fit together like pieces of a jigsaw puzzle. The steps of this dance – the binding mechanism – have been elusive to laboratory experiments due to the difficulty of capturing transient states. Furthermore, the binding mechanism is most directly characterized by analyzing the ensemble of complete, fully continuous pathways between the unbound and bound states along with its kinetics. Simply put, pathways *are* the mechanism.

Atomically detailed molecular dynamics (MD) simulations can, in principle, generate pathways for protein–protein binding processes with high temporal resolution, but are computationally demanding. To our knowledge, only two studies have reported such simulations involving protein–protein binding.^1^,^2^ In one study, the Anton special-purpose supercomputer was used to carry out simulations using the “tempered binding” enhanced sampling approach.^1^ In the other study, a Markov state model^3^ was constructed from an extensive set of discontinuous trajectories to yield discrete states and observables such as rate constants.^2^

Alternative strategies for simulating long-timescale processes employ continuous trajectories to connect states. Such path sampling strategies focus computing effort on the “rare” or infrequent functional transitions between stable states rather than the stable states (*e.g.*, protein conformational transitions upon binding rather than fluctuations within the unbound and bound states). Importantly, the transition pathways are generated without altering the underlying dynamics.^4^ Towards the goal of simulating protein–protein binding processes, the weighted ensemble (WE) path sampling strategy^5^ has enabled the simulation of protein–peptide binding^6^ due to recent advances in its methodology^7^ and software.^8^

The WE strategy exhibits a set of features that distinguish it from other path sampling strategies.^9^ First, rate constants can be computed without a Markov assumption.^7^,^10^ Second, target states (*e.g.* the bound state) need not be strictly defined in advance, permitting refinement of state definitions after the completion of the simulation.^7^ Third, trajectories are queried at fixed time intervals for a resampling procedure, making the WE algorithm interoperable^8^ by enabling the straightforward interfacing with any dynamics engine (*e.g.* Gromacs^11^ and Amber^12^). Furthermore, the WE strategy is rigorous for any type of stochastic dynamics simulation (*e.g.* MD and Brownian dynamics simulations).^13^ Finally, our WESTPA software implementation of the WE strategy scales out to thousands of CPU cores^8^ and can generate pathways and rate constants for long-timescale processes in orders of magnitude less computing time than brute-force simulations (simply running the simulations long enough to capture binding events).^14^–^18^ Moreover, this efficiency increases *exponentially* with the effective free energy barrier of a process.^14^

Here, we applied the WE strategy to orchestrate MD simulation, yielding atomically detailed protein–protein binding pathways and rate constants for the two bacterial proteins, barnase and barstar. The barnase–barstar system has been a classic system for studying protein–protein binding due to the rapid, diffusional-controlled association rate constant (*k*_on_).^19^ The rapid *k*_on_ has been attributed to electrostatic interactions between the negatively charged barnase-binding surface of barstar with the positively charged active site of barnase.^19^ These electrostatic interactions enhance the rate of association by >100-fold relative to the “basal” *k*_on_ for completely hydrophobic versions of the proteins.^16^,^20^ Our study is the most ambitious WE application to date, generating a diverse ensemble of atomistic protein–protein binding pathways with explicit solvation. The resulting simulation not only yields rate constants for the multi-μs timescale binding process (at the effective protein concentration maintained in our simulation), but also resolves short-timescale protein and water dynamics.

## 2. Methods

### 2.1 Weighted ensemble (WE) simulations

#### Overview of the WE strategy

The WE strategy^5^ enhances the sampling of rare events by orchestrating a large number of properly weighted trajectories in parallel and replicating promising trajectories at regular time intervals. Promising trajectories are identified based on a chosen progress coordinate between the initial and target states (*e.g.* unbound and bound states, respectively) that is divided into bins. Importantly, trajectory weights are adjusted according to rigorous statistical rules such that *no bias is introduced into the dynamics*, enabling the calculation of rate constants into any arbitrary state given sufficient sampling. Furthermore, since trajectory weights are independent of the bins, the bins (or even the entire progress coordinate) can be switched during the WE simulation. To maintain steady-state conditions, trajectories that reach the target state are “recycled” by starting new trajectories from the initial state with the same trajectory weights.^5^ Alternatively, WE simulations can be carried out under equilibrium conditions by not recycling any trajectories.

In the present study, the WE strategy was applied under equilibrium conditions to permit the refinement of the target-state definition after completion of the simulation.^7^ Each WE simulation was carried out according to the following steps:

(1) Prepare *M* independent trajectories starting from the initial state conformation (or ensemble of conformations) with each trajectory carrying an equal statistical weight of 1/*M*.

(2) Propagate the dynamics of all *M* trajectories in parallel for a short time interval *τ* that is sufficiently long for at least one of the trajectories to “populate” an empty bin that is further along the progress coordinate.

(3) Apply a resampling procedure, which involves either the replication or pruning of trajectories to maintain the desired number of trajectories in each bin to yield roughly even coverage of configuration space. To replicate a trajectory, start another “child” trajectory from the last state (coordinates, velocities, *etc.*) of the parent trajectory and split the parent trajectory weight evenly among the child trajectories. To prune a trajectory, randomly select one of the lowest-weight trajectories and combine its statistical weight with that of the other trajectory.

(4) Repeat steps 2 and 3 until a sufficient number of trajectories have reached the target state conformation (or ensemble of conformations), *e.g.* to obtain reasonably converged rate constants. The combination of steps 2 and 3 are referred to as a “WE iteration,” *i.e.* the simultaneous propagation of all *M* trajectories for the time interval *τ*, followed by application of the resampling procedure. After *N* iterations, each trajectory has a maximum “molecular time”, or time elapsed, of *N*τ**.

#### Simulation workflow

To generate a diverse ensemble of binding pathways for barnase and barstar, we employed the following workflow:

(1) Run a “preparatory” WE simulation of each isolated protein to extensively sample representative unbound-state conformations of the protein. As demonstrated in a previous study,^6^ the WE strategy is not only efficient at generating rare events, but also in the sampling of protein conformations.

(2) Generate the initial unbound-state ensemble by selecting conformations of each protein from the relevant preparatory simulation according to their statistical weights and constructing 100 unbound pairs of conformations for the two proteins with an extensive range of randomly selected, relative orientations at a minimum separation of 20 Å.

(3) Simulate the binding process by initiating 16 independent trajectories from each of the 100 unique, unbound-state conformations, giving each conformation multiple chances to result in binding pathways. This WE simulation was carried out in two stages to first focus the sampling on formation of the “encounter complex” intermediate and then on rearrangements of encounter complexes to the bound state.

All WE simulations were carried out using the open-source, highly scalable WESTPA software (https://westpa.github.io/westpa).^8^ Full details are provided below.

#### Preparatory simulation of isolated proteins

Representative unbound-state conformations of isolated barnase were sampled by a “preparatory” WE simulation of the protein starting from the heavy-atom coordinates of barnase in the crystal structure of the barnase–barstar complex.^21^ Likewise, unbound-state conformations of isolated barstar were sampled by a preparatory WE simulation starting from the heavy-atom coordinates of barstar in the crystal structure of the complex. Each preparatory simulation was carried out using a one-dimensional progress coordinate that consisted of the heavy-atom RMSD of the protein from its conformation in the crystal structure of the protein–protein complex. This RMSD coordinate was divided into 45 bins, with finely spaced bins every 0.1 Å from 0 to 3.0 Å, more coarsely spaced bins every 0.5 Å from 3.0 to 10 Å, and a single bin for all values ≥10 Å. The simulations were carried out until the probability distributions as a function of the progress coordinate were reasonably converged. In total, *N* = 1200 WE iterations were carried out with a fixed time interval *τ* = 5 ps and a desired number of *M* = 12 trajectories per bin.

#### Generation of the initial unbound-state ensemble

Each member of the initial state ensemble consisted of the two proteins separated by a minimum distance of 20 Å and randomly oriented with respect to one another. Conformations of the pairs of unbound proteins were selected according to their statistical weights from the last WE iterations of the corresponding preparatory WE simulations. A total of 1728 possible unbound pairs were generated and reduced to 100 pairs by assigning trajectories to appropriate bins along the minimum barnase–barstar separation dimension of the two-dimensional binding progress coordinate described below and pruning the lowest-weight trajectories according to the standard WE algorithm.^5^ Each of the resulting 100 pairs was then solvated in dodecahedral boxes of water molecules, equilibrating the solvent as described below under “Propagation of dynamics.”

#### Simulation of the binding process

To simulate the binding process, a single WE simulation was carried out by starting 16 independent trajectories from each of the 100 members of the initial state ensemble, yielding a total of 1600 trajectories with appropriately renormalized trajectory weights. To make optimal use of an available number of CPU cores, the total number of trajectories was fixed at 1600 at all times during the simulation.

The simulation was carried out in two stages. In the first stage, the simulation focused on generating diffusional collisions of the proteins to form the encounter complex until each of the 100 unique unbound-state conformations either formed an encounter complex or did not “survive” due to pruned (terminated) trajectories. In the second stage, the simulation was focused primarily on rearrangements of the encounter complex to the bound state. A two-dimensional progress coordinate was employed throughout the simulation, consisting of (i) the minimum separation between barnase and barstar, and (ii) a “binding” RMSD, which was determined by first aligning on barnase in the crystal structure of the barnase–barstar complex and then calculating the heavy-atom RMSD of the barstar “anchor residues” D35 and D39. We use the term “anchor residues” to refer to residues in a protein that become most buried upon binding their partner protein. Throughout the simulation, the minimum separation coordinate was divided into two bins to partition conformations in which the binding partners were in van der Waals contact (<5 Å) from those conformations in which the binding partners were not in contact (≥5 Å). Furthermore, once the binding partners were in contact at any stage of the simulation, the binding RMSD coordinate was divided into 72 bins with coarsely spaced bins every 1 Å from 10 to 60 Å and more finely spaced bins every 0.5 Å from 0 to 10 Å.

As shown in Fig. S1 in the ESI,† the binning schemes for the two stages of the simulation differed only along the binding RMSD coordinate where the proteins were beyond van der Waals contact (≥5 Å in the minimum separation coordinate). In particular, upon proceeding from the first to the second stage of the simulation, all 72 bins of the binding RMSD coordinate were merged into a single bin. This dramatic reduction in the number of bins – combined with fixing the total number of trajectories at any one time among all bins of the progress coordinate – effectively shifts most of the computing power from sampling the formation of encounter complexes to sampling rearrangements of encounter complexes to the bound state, nearly doubling the number of trajectories for the latter.

To obtain a reasonably converged *k*_on_ with a relative percent error of <50% (Fig. S2 in the ESI†), the binding simulation was carried out for *N* = 650 WE iterations with a fixed interval *τ* = 20 ps, yielding a maximum molecular time (*N*_*τ*_) of 13 ns and an aggregate simulation time of 18 μs. All analysis was performed every *τ* unless otherwise noted.

#### Propagation of dynamics

Dynamics of the WE simulations were propagated using the Gromacs 4.6.7 dynamics engine^11^ with the Amber ff03* force field^22^ and TIP3P water model.^23^ Heavy-atom coordinates for initial models of the unbound proteins and native complex were extracted from the crystal structure of the barnase–barstar complex (PDB code: ; 1BRS).^21^ Hydrogen atoms were added to each model using ionization states present in solution at pH 7. Each system was immersed in a sufficiently large dodecahedron box of explicit water molecules to provide a minimum 12 Å clearance between the solutes and box walls for the unbound states in which the binding partners were separated by 20 Å. A total of 31 Na^+^ and 29 Cl^–^ ions were included to neutralize the net charge of the protein system and to yield the experimental ionic strength (50 mM).^19^ The entire simulation system consisted of ∼100 000 atoms.

Prior to carrying out WE simulations, the systems were first subjected to energy minimization and then two stages of equilibrating the solvent while applying harmonic constraints to the proteins with a force constant of 10 kcal mol^–1^ Å^–2^. During the first stage, the system was equilibrated for 20 ps at constant temperature (25 °C) and volume. During the second stage, the system was equilibrated for 1 ns at constant temperature (25 °C) and pressure (1 atm). Since the WE strategy requires stochastic dynamics,^13^ a stochastic thermostat was used, *i.e.* the velocity rescaling thermostat,^24^ with a coupling constant of 0.1 ps. Pressure was maintained using a weak Berendsen barostat^25^ with a coupling constant of 0.5 ps. To enable a 2 fs time step, bonds involving hydrogens were constrained to their equilibrium values using the LINCS algorithm.^26^ van der Waals interactions were switched off smoothly between 8 and 9 Å along with the application of a long-range analytical dispersion correction to energy and pressure. Real-space electrostatic interactions were truncated at 10 Å and long-range electrostatic interactions were calculated using particle mesh Ewald summation.^27^

#### State definitions

**Fig. 1:**
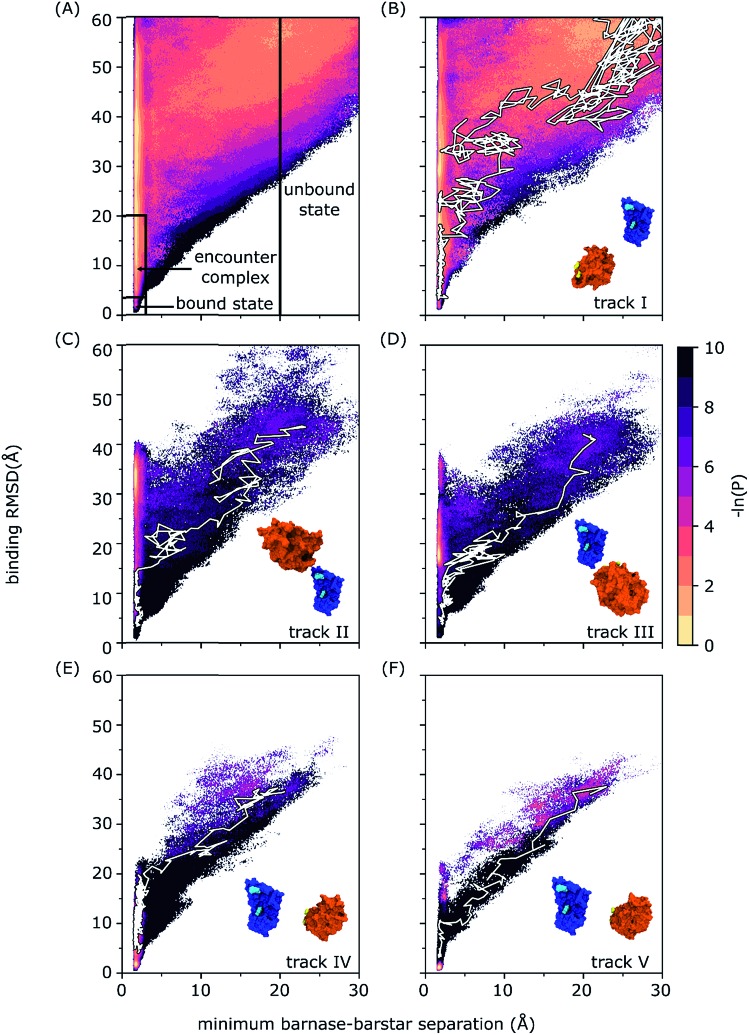
Probability distributions as a function of the WE progress coordinate for (A) all simulation data with definitions of the unbound state, encounter complex, and bound state delineated by solid black lines and (B–E) tracks I–V. In each of the panels B–E, a snapshot of the initial unbound-state conformation is included with molecular surface representations of barnase and barstar in blue and orange, respectively, barnase anchor residues (K27 and R59) are highlighted in cyan, and barstar anchor residues (D35 and D39) are highlighted in yellow; a representative binding pathway along the corresponding probability distribution is highlighted in white. The probability distribution of each binding track was normalized according to the total probability of that track. The color scale represents –ln *P* where *P* is the probability density calculated as the sum of appropriate trajectory weights.

For all analysis, the definitions of key states were determined from the probability distribution yielded by the WE binding simulation as a function of the progress coordinate (Fig. 1A). The initial unbound state was defined as any conformation having a minimum separation of ≥20 Å between the proteins. The encounter-complex intermediate was defined to include only non-native complexes that had a sufficiently long survival time to proceed to the native complex, *i.e.* binding RMSD (defined above) of ≥4 Å and ≤20 Å for D35 and D39 of barstar and a minimum separation of ≤3 Å between the proteins. The target bound state was defined as having a binding RMSD ≤ 3.5 Å for D35 and D39 of barstar and a minimum separation of ≤3 Å between the proteins.

### 2.2 Calculation of rate constants and percentage of productive collisions

To calculate rate constants between states A and B from an equilibrium WE simulation, the equilibrium set of trajectories is first decomposed into two steady states: a forward steady state consisting of trajectories more recently in A than B and the reverse steady state with those most recently in B than A.^7^ Rate constants can then be calculated from the steady state in the relevant direction. Similarly, equilibrium state populations can be decomposed into the component steady-state populations.

In the present study, our equilibrium set of trajectories was decomposed into the “binding” and “unbinding” steady states. Since our WE simulation was intended to enhance the sampling of binding trajectories, we did not generate a large set of unbinding trajectories and therefore focused exclusively on the kinetics of the binding steady state.

In addition to the association rate constant *k*_on_ for the overall two-step binding process, we calculated the rate constant *k*_1_ for the first step involving the formation of the encounter complex and the rate constant *k*_2_ for the second step involving the rearrangement of the encounter complex to the bound state. For *k*_on_, states A and B are the unbound and bound states, respectively; for *k*_1_, the unbound state and encounter complex; and for *k*_2_, the encounter complex and bound state.

The bimolecular rate constants, *k*_on_ and *k*_1_, and unimolecular rate constant *k*_2_ were calculated using the following equations:
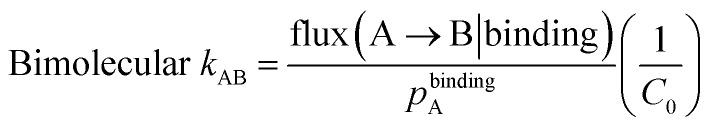
 
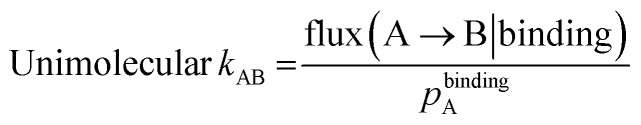
where flux(A → B|binding) is the conditional flux of probability carried by trajectories in the binding steady state, given that the trajectories originated in state A and arrived in state B at any point in the simulation; *p*bindingA is the steady-state population of state A for the binding steady state, *i.e.* sum of the statistical weights of trajectories more recently in state A than in state B; and *C*_0_ is the effective protein concentration maintained in the simulation (1.7 mM), calculated as 1/(*N*_A_*V*) where *N*_A_ is Avogadro's number and *V* is the volume of the simulation box (956 Å^3^).

The conditional probability flux flux(A → B|binding) and its normalization by *p*bindingA focuses the rate constant calculation exclusively on the binding steady state.^7^ The rate constant *k*_1_ was calculated using the last 100 WE iterations of the first stage of the simulation while *k*_2_ and *k*_on_ were calculated using the last 100 WE iterations of the second stage of the simulation.

The percentage of productive collisions (*i.e.*, encounter complexes that succeed in rearranging to the bound state) was calculated, as done before,^6^,^28^ according to the following equation:
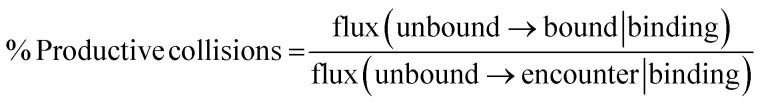
where flux(unbound → bound|binding) and flux(unbound → encounter|binding) are the conditional fluxes for the unbound state → bound state transition and unbound state → encounter complex transition, respectively, from the binding steady state. The former was calculated using the last 100 WE iterations of the second stage of the simulation and the latter was calculated using the last 100 WE iterations of the first stage of the simulation.

Uncertainties in rate constants and the percentage of productive collisions represent 95% confidence intervals. The former was estimated using a Monte Carlo blocked bootstrapping technique.^5^,^29^ The latter was estimated by first calculating the error in the fluxes using the blocked bootstrapping technique and then propagating the error.

The blocked bootstrapping technique involves first bootstrapping over the per-iteration conditional probability flux flux(A → B|binding) to determine the correlation time *t*_c_ of flux(A → B|binding) representing the maximum lag time for which the autocorrelation of flux was statistically significant. The flux values were then averaged over blocks of length *t*_c_, and then a second bootstrap over those blocked average values was used to determine the confidence interval on the flux values.

In our case, a bootstrap using 1000 datasets drawn (with replacement) from all unbound-to-bound flux values over the latter half of the simulation indicated that there was no significant autocorrelation in the unbound-to-bound flux at a lag of *τ* at a 95% confidence level (Fig. S3 in the ESI†). Therefore, the uncertainties in the unbound-to-bound flux assume that the flux values from all WE iterations are statistically independent to 95% confidence.

The number of statistically independent binding events was determined similarly by bootstrapping over the number of arrivals at the bound state per WE iteration.

### 2.3 Maps of ligand entry point distributions and conformation space networks

As done by Dickson and Lotz,^30^ we generated spherical maps of ligand entry points and constructed conformation space networks to visualize the evolution of various properties along the ensemble of simulated binding pathways.

Spherical maps were constructed by projecting the probability distribution of ligand entry points for diffusional collisions of the barstar ligand and barnase receptor onto a unit sphere centered on the barnase receptor. The receptor was rotated such that W44 of barnase is aligned with the *z*-axis and R59 of barnase is aligned with the *y*-axis. The probability distribution was generated by creating a histogram using 30 bins and trajectory weights at each ligand entry point.

Conformation space networks were constructed by first applying the KCenters clustering algorithm for each of the 203 binding events and then generating force-directed layouts with the resulting 2000 clusters. The clustering was carried out using a Canberra distance metric as implemented in the MSMBuilder software package^31^ and a feature vector that consisted of the WE progress coordinate used for the binding simulation. Force-directed layouts were generated using the ForceAtlas 2 layout algorithm,^32^ as implemented in the Gephi 0.9.2 software package.^33^ Each node in the layouts represents a cluster center and the edges between nodes represent observed transitions between each cluster. The size of each node is proportional to the total statistical weight over all conformations in the corresponding cluster and colored according to the weighted average of the property of interest (*e.g.* extent of protein desolvation and percent burials of particular residues) over all conformations of that cluster.

To monitor the extent of protein desolvation during the binding process, we tracked the number of water molecules *N*_w_ within 6 Å of each protein to encompass the first two solvation shells. We then calculated a “percent solvation” by dividing the average *N*_w_ in a particular conformation by the average *N*_w_ in the ensemble of unbound-state conformations.

Percent burials upon binding for barstar residues, D35, D39, W38, and W44, were calculated as (SASA in the selected configuration)/(average SASA in the unbound state) × 100% where the SASA is the solvent accessible surface area and calculated using the Shrake and Rupley algorithm^34^ as implemented in MDTraj Python library.^35^

### 2.4 Calculation of pairwise residue contact maps

To identify kinetically important residues for the binding process, we searched for the most probable intermolecular residue contacts in the transition path ensemble (TPE), which consists of only the transient states along the productive binding pathways between stable states. These so-called “transition paths” begin where the trajectory last exits the initial unbound state and end where the trajectory first enters the bound state. A pair of residues was considered to be in contact if any of their heavy atoms were ≤4.5 Å of each other. The probability of forming a pairwise residue contact in the TPE was calculated by summing over the statistical weights of all TPE conformations where the two residues are in contact; the weight of each TPE conformation was calculated by summing over the weights of its successful child trajectories.

### 2.5 Calculation of sidechain conformational entropy per residue

The sidechain conformational entropy *S*_X_ of each residue in a given state X (*i.e.* unbound state, encounter complex, or bound state) was calculated using the following:
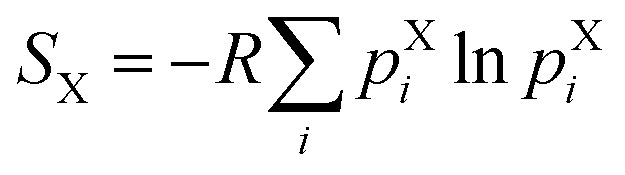
where *R* is the ideal gas constant and *p*X*i* is the probability of observing a particular set *i* of *χ* angles for the sidechain of the residue in state X in the WE simulation. To generate a histogram of each probability distribution, different numbers of bins were tested, ranging from 15 to 35 bins. Only qualitative conclusions were drawn from each histogram and these conclusions were consistent for the different numbers of bins.

### 2.6 Calculation of percent occupancies of interfacial crystallographic water positions

The percent occupancy of each of the nine interfacial, crystallographic water positions was calculated for the ensemble of bound-state conformations by summing over the probabilities of all trajectories in which a water molecule occupies one of the nine positions, forming hydrogen bonds with the corresponding residues in each of the two proteins. Trajectory probabilities were normalized by the population of the bound-state ensemble and included in the sum of probabilities if a given position is occupied at any point within each WE iteration of fixed length *τ* = 20 ps. Hydrogen bond formation was monitored every ps and defined as having a donor–acceptor distance of ≤3 Å and a donor–acceptor angle of ≥90° using the MDAnalysis Python library.^36^

## 3. Results and discussion

### 3.1 Overall binding mechanism and kinetics

Our WE simulation was successful in generating a large diverse ensemble of fully-continuous, atomically detailed binding pathways for the barnase and barstar proteins in explicit solvent. In particular, 43 of the 100 unique, unbound-state conformations altogether resulted in 203 statistically independent binding pathways (see Methods) within 30 days using 1600 CPU cores at a time on XSEDE's Stampede supercomputer, yielding 18 μs of aggregate simulation time.

Results reveal a two-step binding process in which an “encounter complex” intermediate (Fig. 1A) is first formed, followed by rearrangement of the encounter complex to the native, bound state. While only 4% of the aggregate simulation time yielded successful binding pathways, 81% resulted in diffusional collisions and 35% resulted in the formation of encounter complexes, which are defined here as productive end points of diffusional collisions that eventually rearrange to the bound state. We note that all percentages reported in this study represent WE weighting.

The extensive sampling provided by the WE strategy enabled not only the computation of rate constants, but the percentage of diffusional collisions that were productive, *i.e.* eventually resulting in the bound state. Among all diffusional collisions, only 11 ± 5% were productive, which is similar to the computed percentage of productive collisions (∼10%) for another diffusion-controlled protein binding process (*i.e.* for the MDM2 protein and p53 peptide).^6^ Importantly, the computed association rate constant *k*_on_ for barnase and barstar [(2.3 ± 1.0) × 10^8^ M^–1^ s^–1^] is within error of experiment [(2.86 ± 0.7) × 10^8^ M^–1^ s^–1^],^19^ demonstrating that WE sampling can be useful for validating the force field in modeling long-timescale properties, especially kinetics observables such as rate constants. As shown in Table 1, the computed rate constant for formation of the encounter complex *k*_1_ [(1.8 ± 0.2) × 10^9^ M^–1^ s^–1^] is approximately equal to the *k*_on_ and the computed rate constant *k*_2_ for rearrangement of the encounter complex to the bound state is relatively fast [(2.7 ± 0.5) × 10^10^ s^–1^]. The rate-limiting step for the binding process is therefore the diffusion-controlled formation of the encounter complex. The rate constant *k*_1_ for this initial step is on the order of the Smoluchowski limit (∼5 × 10^9^ M^–1^ s^–1^) despite orientational constraints due to electrostatic interactions between the proteins.^37^

**Table 1:** Computed rate constants and 95% confidence intervals for the barnase–barstar binding process

Process	Rate constant	Value
Unbound state → encounter complex	*k*_1_ (M^–1^ s^–1^)	1.8 ± 0.2 × 10^9^
Encounter complex → bound state	*k*_2_ (s^–1^)	2.7 ± 0.5 × 10^10^
Unbound state → bound state	*k*_on_ (M^–1^ s^–1^)	2.3 ± 1.0 × 10^8^
	Experimental *k*_on_ (ref. 19) (M^–1^ s^–1^)	2.86 ± 0.67 × 10^8^

### 3.2 Binding pathways and the free energy landscape

Our results provide direct confirmation of a “funnel-like” free energy landscape for the diffusion-controlled, protein–protein binding process of barnase and barstar. In particular, once the proteins collide productively to form an encounter complex, the rearrangement of the encounter complex to the bound state is largely downhill and therefore fast relative to the rate-limiting formation of the encounter complex (Fig. 1A). The idea of a funnel near the protein binding site has been previously proposed by theoretical studies.^38^–^42^ In particular, the existence of a binding funnel rationalizes how protein–protein associations can occur >10^3^ times faster than would be expected from the docking of spherical models that have specific orientational constraints (∼10^3^ M^–1^ s^–1^).^43^

Multiple binding pathways were generated by our simulation. These pathways fall along five separate “tracks” (tracks I to V) that each originated from a different configuration of the unbound state and are therefore independent with no common trajectory segments (Fig. 1B–F). The tracks vary in the extent to which the binding partners must rotate relative to each other in order to collide and form productive encounter complexes. Track I (Fig. 1B) is the most indirect binding track with the binding interfaces of the two proteins pointing away from each other thereby requiring the largest extent of rotations of both binding partners to collide productively. Tracks IV and V (Fig. 1E and F) are the most direct binding tracks, requiring the smallest extent of rotations. Despite the fact that the unbound configurations for tracks IV and V are very similar, track V yielded less direct pathways than track IV to forming the encounter complex.

The most probable binding track is the most indirect (track I, Fig. 1B), accounting for almost the entire probability distribution that was sampled by our simulation as a function of the WE progress coordinate (Fig. 1A). Furthermore, the distribution of event duration times, or barrier crossing times, is essentially identical for the most indirect track and the full set of binding pathways with the most probable event duration being 6.7 ns (Fig. S4 in the ESI†). The fact that the most indirect binding pathways are the most probable pathways indicates that the binding interfaces of the partner proteins are not highly likely to be pointing directly at each other in their unbound states such that the proteins must rotate in order to productively collide to form encounter complexes that eventually rearrange to the native complex. Furthermore, such rotations are likely due to long-range electrostatic steering of the binding interfaces towards each other, as demonstrated by a previous simulation study with rigid protein models.^20^

### 3.3 Features of the most probable binding track

**Fig. 2:**
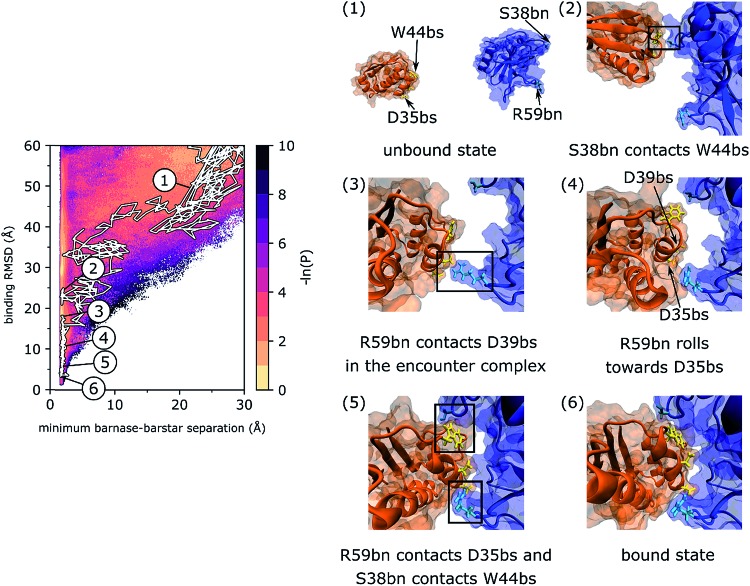
A representative, continuous binding pathway of the most probable track (track I). On the left, the pathway is highlighted in white along the probability distribution of the WE progress coordinate. The simulation was initiated from (1) an unbound state in which barnase (blue) and barstar (orange) were separated by 20 Å and their binding interfaces were pointing away from each other. Binding interface residues of barnase, S38bn and R59bn, are shown in cyan. Binding interface residues of barstar, D35bs and D39bs, are shown in yellow. Diffusional collision of the binding partners initially formed transient W44bs-S38bn contacts (2), which then dissociated upon forming D39bs-R59bn contacts in the encounter complex state (3). Eventually, R59bn rolled along the molecular surface of barstar to form contacts with D35bn while reforming W44bs-S38bn contacts (5). The formation of these contacts at opposite edges of the binding interfaces facilitated rearrangement of the encounter complex to the native, bound state (6). This binding pathway is also illustrated by Movie S1 in the ESI.†

As shown in Fig. 2, a representative pathway along the most probable binding track (track I) involves interactions of barstar (bs) with two loops in barnase (bn) that flank its binding interface: one loop that includes S38bn and the RNA recognition loop that includes R59bn. Upon collision of the unbound proteins, transient contacts between S38bn and W44bs are initially formed, but eventually dissociate when R59bn forms contacts with D39bs. The two proteins then “roll” along each other's binding interfaces until R59bn forms contacts with D35bs and S38bn reforms contacts with W44bs. These two sets of contacts fasten the opposite edges of the barnase binding interface thereby facilitating rearrangement of the encounter complex to the bound state. The rolling of partner proteins along each other's molecular surfaces has also been observed in previous simulation studies of protein binding processes as a means of rearranging encounter complexes to native, bound states.^44^,^45^

Interactions of barstar with the two barnase loops, *i.e.* with S38bn and R59bn, have also been identified using Markov state models that were constructed from simulations with a different force field (Amber ff99SB-ILDN^46^ and the TIP3P water model^23^).^2^ In particular, a small percentage (5%) of the transition-state ensemble for binding consisted of interactions with S38bn while the majority (95%) of the same ensemble consisted of interactions with R59bn. It is worth noting that the Markov state model necessitates the use of a significant lag time (in that study, 110 ns), which fundamentally limits the ability to resolve features of the binding mechanism below such timescales (*e.g.* short-timescale protein and water dynamics). Thus, while the aggregate simulation time of our WE simulation is <1% of that used for the Markov state model, our simulation not only generates continuous pathways, but also resolves short-timescale mechanistic details such as the “rolling” of R59bn along the binding interface of barstar during the rearrangement of the encounter complex to the bound state.

Given the subtle differences that exist between the crystal structures of barnase and barstar in their unbound^47^,^48^ and bound states,^21^ it might appear that the barnase–barstar complex forms by rigid-body association. However, our simulations reveal a more dynamic view of the two proteins during their molecular dance towards a final “embrace” to form the bound state (Movie S1 in the ESI†). Consistent with an NMR study,^49^ many of the protein sidechains are highly dynamic on the ps timescale with the dynamics changing significantly upon binding.

### 3.4 Kinetically important interactions

**Fig. 3:**
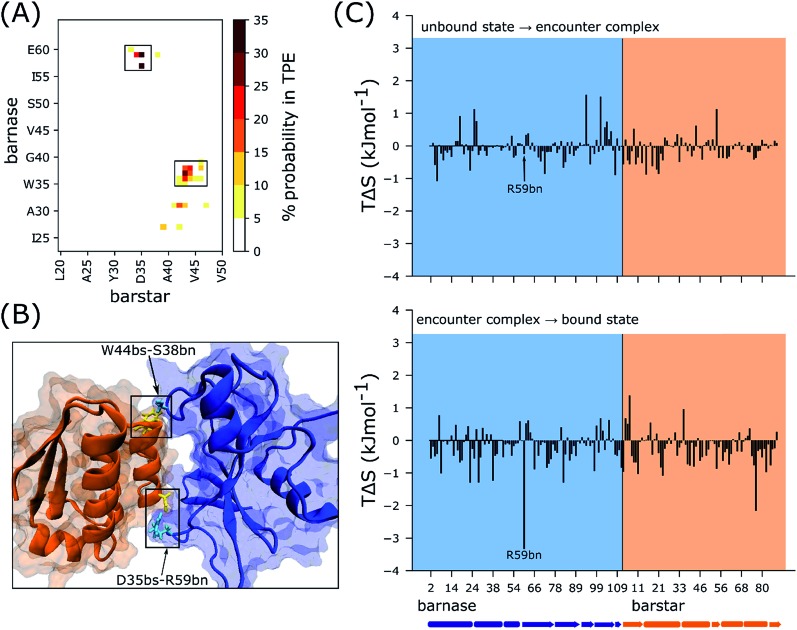
(A) Map of pairwise residue contacts formed in the encounter complex ensemble. The color bar represents the percent probability of forming a given pair of residue contacts in the transition path ensemble (TPE). (B) Locations of the most kinetically important residue contacts indicated in the crystal structure of the native complex of barnase (blue) and barstar (orange) with R59bn and S38bn in cyan and D35bs and W44bs in yellow. (C) *T*Δ*S* per residue where Δ*S* is the change in sidechain conformational entropy of the residue upon forming the encounter complex (top) and upon rearrangement of the encounter complex to the bound state (bottom). Barnase and barstar residues are indicated by the blue and orange shaded regions, respectively. Secondary structure elements are indicated below the *x*-axis. As highlighted, R59bn is the residue with the largest *T*Δ*S* upon rearrangement of the encounter complex to the bound state. Each histogram shown here was generated using 25 bins (see Methods).

To identify kinetically important interactions for the binding process, we constructed a map of probabilities of forming each possible pair of intermolecular residue contacts in the transient states that comprise the transition path ensemble (TPE; see Methods). As shown in Fig. 3A and B, the most probable contacts are D35bs-R59bn and W44bs-S38bs, which are formed 34% and 23% of the time, respectively. As mentioned above, these two contacts fasten both edges of the barnase binding interface in the encounter complex and in doing so, reduce the sidechain conformational entropy of R59bn more than any other residue upon rearrangement of the encounter complex to the bound state (Fig. 3C).

**Fig. 4:**
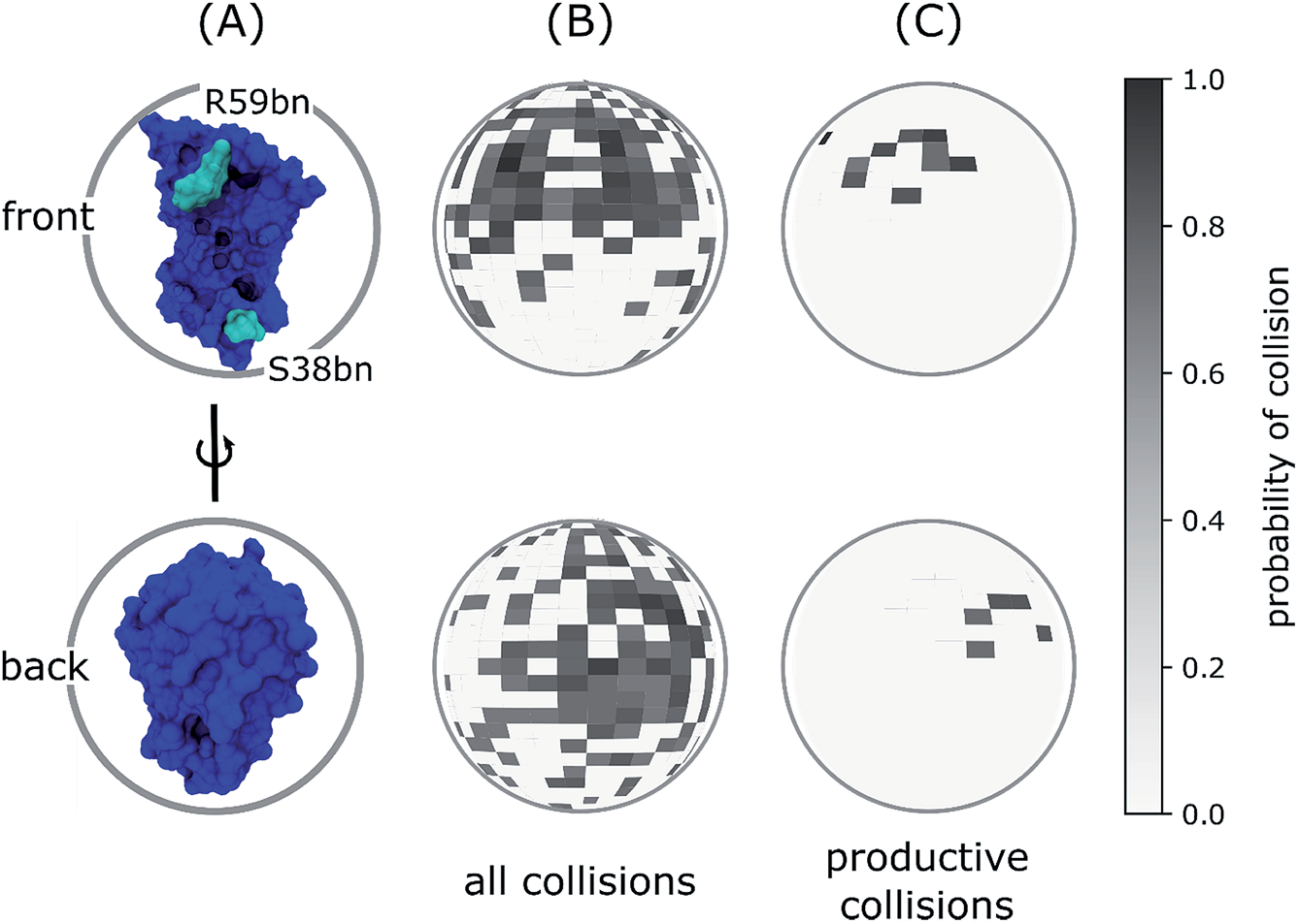
Spherical maps of ligand entry point distributions for the barstar ligand in diffusional collisions with the barnase receptor. (A) Reference orientations of barnase (blue) for ligand entry point distributions in panels B and C where each probability distribution is projected onto a unit sphere centered on barnase in one of two orientations: the front view corresponding to a “head-on” view of the barnase binding interface and the back view corresponding to a 180° rotation around the vertical axis of the front view. (B) Ligand entry point distributions for all diffusional collisions. (C) Ligand entry point distributions for only productive collisions that form encounter complexes, which subsequently rearrange to the bound state. As shown in panel C, the most probable entry points are in the vicinity of R59bn (cyan in panel A), which lies at one edge of the barnase binding interface, as opposed to S38bn, which lies at the other edge.

The importance of R59bn for the binding kinetics is also evident from an analysis of the encounter complex ensemble. In particular, among the diverse relative orientations of the binding partners that resulted in collisions to form encounter complexes, productive collisions generally involved contacts with R59bn or other residues in its vicinity (Fig. 4). Consistent with this result, previous simulation with rigid protein models have demonstrated that the RNA recognition loop, on which R59bn resides, may electrostatically steer barnase and barstar towards one another in relative orientations that are productive for forming the encounter complex.^20^,^50^

R59bn has been identified by previous studies to be the most kinetically important residue for the barnase–barstar binding process. In particular, the experimental *k*_on_ is reduced by >9-fold upon mutation of R59bn to an alanine.^51^ In addition, R59bn formed intermolecular contacts in the majority of transition-state conformations that were characterized by a simulation study that involved the construction of Markov state models.^2^

Interestingly, the barstar anchor residues, D35bs and D39bs, are not only the most buried upon binding barnase, but buried the earliest among barstar residues with D35bs burying before D39bs (Fig. S5A and S5B in the ESI†). In addition, both Trp residues at the barnase binding interface, W38bn and W44bn, are buried upon forming the encounter complex with W44bn burying before W38bn (Fig. S5C and S5D in the ESI†). Thus, changes in the intrinsic Trp fluorescence of barnase might result from the formation of the encounter complex as well as the formation of the native complex in time-resolved experiments.

### 3.5 Evolution of solvent configuration during the binding process

**Fig. 5:**
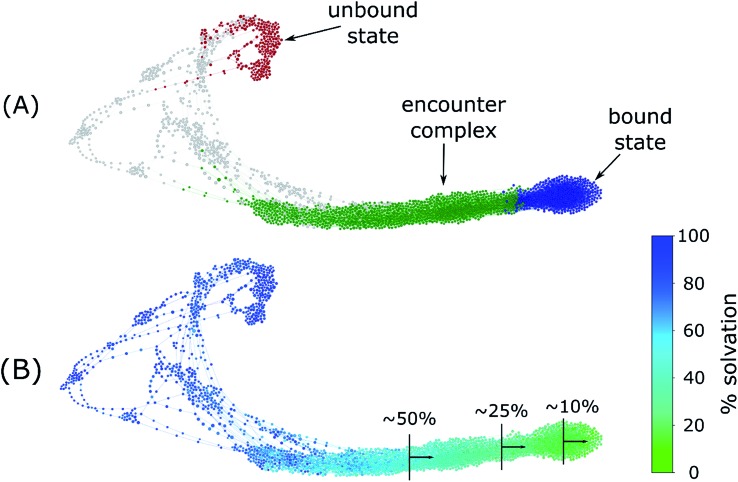
Conformational space networks of barnase–barstar binding pathways colored according to (A) state definitions used for calculations of rate constants, and (B) percent solvation in a given cluster of conformations (see Methods).

To determine when the two proteins undergo desolvation of their binding interfaces before forming the native complexes, we monitored the percent solvation of each conformation relative to the unbound state, tracking the number of water molecules within 6 Å of each protein (see Methods). We then generated a conformational space network to visualize the various binding tracks and colored this network according to the minimum percent solvation thereby detecting *any* instance of desolvation. As shown in Fig. 5, protein desolvation occurs in the late stages of the binding process in our simulations. In particular, the two proteins undergo the greatest extent of interface desolvation during the rearrangement of the encounter complex to the native complex. This result is consistent with an experimental study in which the characterization of the transition state for the rearrangement of the encounter complex to the native complex revealed that most of the interface desolvation has not yet occurred based on a low activation entropy.^52^

Notably, our simulations revealed that during the binding process, water molecules from the bulk solvent eventually occupy the positions of all but one of the nine interfacial crystallographic water molecules that bridge hydrogen bonds between barnase and barstar in the native complex.^21^ As shown in Table 2, the eight positions are occupied 4% to 48% of the time, frequently exchanging with water molecules from the bulk solvent. For example, in the representative pathway that is illustrated in Fig. 2, a given position was occupied for ≤1.3 ns by the same water molecule. The occupancy of these positions is an encouraging validation of the force field and water model – particularly since the simulations were started from the unbound state. Furthermore, these results suggest that the interfacial water molecules in the crystal structure of the native complex are present in solution as well as the crystal environment. Finally, our simulation identified water molecules in the bound-state ensemble that are not resolved in the crystal structure of the native complex and bridge hydrogen bonds between residues that we have identified as kinetically important (Fig. S6 in the ESI†).

**Table 2:** Percent occupancies of solvent in the simulated bound-state ensemble at positions of crystallographic waters that bridge hydrogen bonds between residues in barnase (bn) and barstar (bs). Crystallographic waters are listed in order of top to bottom along the corresponding positions (red spheres) at the binding interfaces of barnase (blue) and barstar (orange)

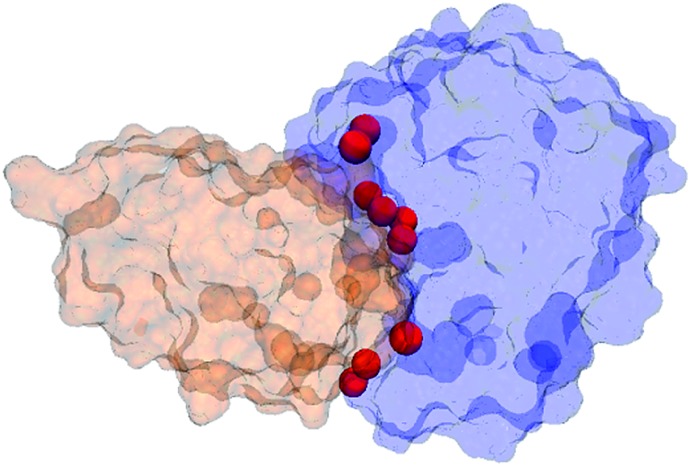			
Solvent	Solvent ASA (A^2^)	Residues bridged by hydrogen bonds of the water molecule	% Occupancy in the simulated bound-state ensemble
Wat128	0	K62bn/Y103bn – D35bs	17
Wat22	0	K62bn/N58bn – D35bs	22
Wat29	0	R59bn – D35bs	21
Wat14	0	E73bn – D35bs	0
Wat48	3	I55bn/E73bn – W38bs	33
Wat33	0	K27bn/E73bn – D39bs	48
Wat155	1	R83bn – G43bs	6
Wat36	9	S38bn – V45bs	11
Wat93	17	S38bn – Y47bs	4

### 3.6 Choice of progress coordinate for protein binding processes

As described in Methods, our WE simulation of the protein–protein binding process employed a two-dimensional progress coordinate consisting of (i) the minimum separation distance between barnase and barstar, and (ii) the binding RMSD of the two barstar “anchor” residues, D35 and D39. The first dimension was intended to partition conformations into those in which the proteins were not in contact and those in which the proteins had collided. This partitioning ensured the formation of encounter complexes with large binding RMSD values, which was instrumental in improving the statistical precision of the rate constants involving encounter complexes (*k*_1_ and *k*_2_) and the percentage of productive collisions. The second dimension was intended to generate all types of collisions (productive and nonproductive) starting from a diverse set of relative orientations of the binding partners in their unbound states and to distinguish between the nonnative encounter complex from the native bound state.

Although the RMSD of the entire native complex has been previously found to be a poor descriptor for monitoring the progress of protein–protein association pathways,^20^ we have demonstrated that the RMSD can be an effective progress coordinate if the deviations are calculated for only the ligand after alignment of the protein receptor. At large ligand–receptor separations, this “binding” RMSD functions as a distance metric and at shorter separations, as a metric of both distance and relative orientations of the binding partners with respect to those of the bound state. For both this study and our previous atomistic WE study of protein–peptide binding,^6^ we have focused on taking the binding RMSD of the minimal set of key ligand residues for binding – the ligand “anchor” residues, which are the residues that become the most buried upon binding the protein receptor. Such anchor residues have been proposed to smooth out the binding process by avoiding kinetically costly structural rearrangements.^53^

### 3.7 Applicability of our strategy to simulation of other receptor–ligand binding processes

Our WE strategy is a general one that can enable the generation of atomistic pathways and characterization of kinetics for the binding process of any receptor–ligand system in which the receptor and ligand are largely preorganized for binding, including other protein–protein systems, protein–drug ligand systems, and host–guest systems. In cases where the folding of a ligand occurs only upon binding its receptor, it may be sufficient to include an additional dimension to the progress coordinate that monitors the folding of the ligand.^6^ The only prerequisite is that the structure of the receptor–ligand complex is available, *e.g.* from X-ray crystallography or NMR spectroscopy. Based on the structure of the complex, initial coordinates can be extracted for sampling the ensemble of unbound conformations for each binding partner and the ligand “anchor” residues that become the most buried upon binding can be identified and used for monitoring the extent of binding based on a “binding” RMSD progress coordinate.

We note that the main limitation of WE and related strategies is that the free energy barriers to be surmounted may be orthogonal to the selected progress coordinate. In principle, however, if the progress coordinate captures the slowest relevant motion, then faster, correlated coordinates will also be captured.^9^ Furthermore, bins and progress coordinates can be switched “on the fly” during a WE simulation since trajectory weights are independent of the bins.^13^

A future goal for the development of WE strategies is to generate an even greater diversity of binding pathways and thereby greater precision in computed rate constants. Promising strategies are the use of semi-automated binning schemes to more extensively cover configurational space (*e.g.* adaptive construction of bins using Voronoi procedures^13^) and the improvement of schemes for replication and pruning of trajectories to prioritize the generation of binding pathways from distinct unbound-state conformations.

## 4. Conclusions

In closing, we have demonstrated the power of the weighted ensemble (WE) strategy^5^ in enabling explicit-solvent MD simulation of a protein–protein binding process. Our simulation involves the barnase–barstar system, a prototypical system for studying diffusional-controlled protein–protein binding processes. Results provide a number of insights regarding the binding mechanism that cannot be obtained by laboratory experiments.

First, our simulation provides atomically detailed views of the binding pathways, including states that are too transient to be captured by experiment. Among the diverse ensemble of binding pathways (203 independent pathways), the most probable pathways were the most indirect, requiring the largest extent of rotations of the partner proteins in order to collide productively to form “encounter complex” intermediates. The rotations likely result from long-range electrostatic steering of the binding interfaces towards each other. Upon forming the encounter complex, the subsequent rearrangement of the encounter complex to the bound state involves “rolling” of the proteins along each other's binding interfaces.

Second, our simulation directly yields rate constants for individual steps of the binding process while time-resolved experiments can measure rate constants for only the *overall* binding process. In particular, the simulation revealed a two-step binding process in which the formation of the encounter complex is rate-limiting followed by the relatively fast rearrangement of the encounter complex to the bound state. Notably, our simulation provides direct confirmation that the free energy landscape for protein–protein binding can be “funnel-like” toward the native, bound state. It is also worth noting that the extensive sampling of the WE strategy was useful for validating the force field in modeling long-timescale binding kinetics, yielding a computed k_on_ that is within error of experiment.^19^ Furthermore, WE sampling enabled the calculation of the percentage of productive collisions with 11 ± 5% of all diffusional collisions being productive, *i.e.* eventually resulting in the bound state.

Third, our simulation identified the most kinetically important interactions for the binding process. These interactions, which involve barnase residues, R59bn and S38bn, fasten opposite ends of the binding interface prior to rearrangement of the encounter complex to the bound state.

Finally, short-timescale solvent dynamics during the binding process were resolved in our simulation, including the rolling of the two proteins along each other's surfaces during the rearrangement of the encounter complex to the bound state. Throughout the binding process, our simulations revealed more dynamic sidechain motions of the proteins than expected from the subtle differences between crystal structures of the corresponding unbound and partner-bound states.^21^,^48^,^54^ In addition, desolvation of the protein binding interfaces was found to occur during the late stage in the binding process just prior to rearrangement of the encounter complex to the native complex. Once the bound state was reached, all but one of the nine positions of interfacial crystallographic water molecules that bridge hydrogen bonds between barnase and barstar were occupied by water molecules that originated from the bulk solvent.^21^

Taken together, our WE simulation provides direct views of pathways at an unprecedented level of detail as well as the necessary sampling to validate current simulation models, especially in the calculation of kinetics observables. Given that the simulation could now be completed within 10 days on a GPU using 16 NVIDIA Tesla P100 GPUs at a time, WE-enabled atomistic simulations of multi-μs protein binding processes are now practical on typical resources. Furthermore, our simulation strategy is a general one that can be applied to any receptor–ligand complex in which the receptor and ligand and largely preorganized for binding. Thus, the WE strategy and others like it have great promise in providing insights involving binding kinetics for a variety of research areas, including biophysics, catalysis, protein engineering, and material design.

## Conflicts of interest

There are no conflicts to declare.

## Supplementary Material

Supplementary information
