# Supplementary material for: Protein–protein binding pathways and calculations of rate constants using fully-continuous, explicit-solvent simulations
Source: Chem Sci. 2018 Dec 27;10(8):2360–72. doi: 10.1039/c8sc04811h (PMC6385678; doi:10.1039/c8sc04811h)
Supplement: Supplementary file 1 [file SC-010-C8SC04811H-s001.pdf]

Electronic Supplementary Material (ESI) for Chemical Science.
This journal is © The Royal Society of Chemistry 2019

Protein-protein binding pathways and calculations of rate constants using fully-continuous, explicit-solvent simulations

Ali S. Saglam and Lillian T. Chong

SUPPORTING INFORMATION

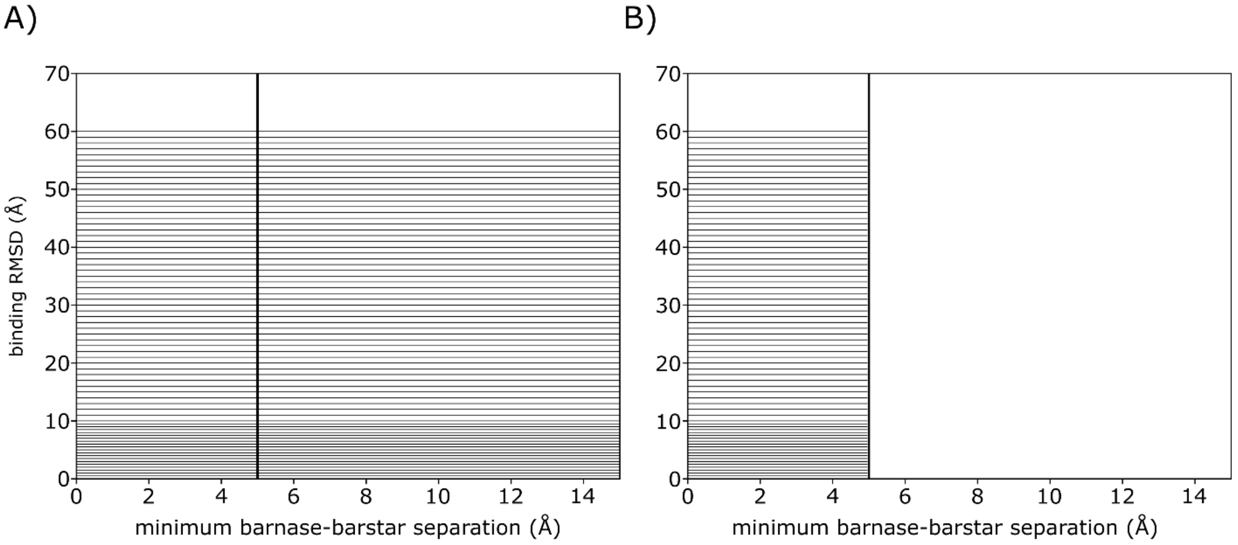

Fig. S1 Binning schemes used for the two-dimensional WE progress coordinate in A) the first stage of the binding simulation to focus the sampling on diffusional collisions to form encounter complexes, and B) the second stage of the binding simulation to focus the sampling on rearrangements of encounter complexes to the bound-state ensemble. The scheme in panel B) differs from the scheme in panel A) by the merging of 72 bins along the RMSD coordinate (y-axis) to a single bin in the region where the minimum barnase-barstar separation (x-axis) is beyond van der Waals contact (≥5 Å). For both stages of the binding simulation, the minimum barnase-barstar separation was divided into two bins to separate conformations with the binding partners in van der Waals contact (<5 Å) and not in contact (≥5 Å); furthermore, in the region where the minimum barnase-barstar separation <5 Å, the RMSD coordinate was divided into 72 bins at any point in the simulation with coarsely spaced bins every 1 Å from 10 to 60 Å and more finely spaced bins every 0.5 Å from 0 to 10 Å.

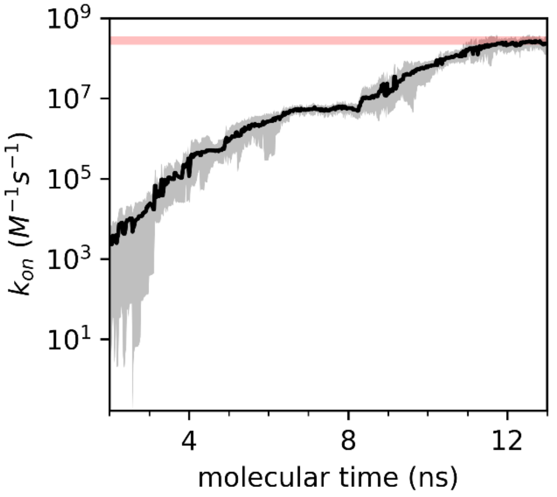

Fig. S2 Evolution of the computed k_{on} for barnase-barstar association as a function of molecular time. The molecular time is defined as Nτ where N is the number of iterations in the weighted ensemble simulation and τ is the fixed time interval (20 ps) of each iteration. Data shown in black is a running average of the computed k_{on} over 100 WE iterations (2 ns) along with and 95% confidence intervals (gray shaded region). The experimental k_{on}[1] along with 95% confidence interval is indicated by the pink shaded region.

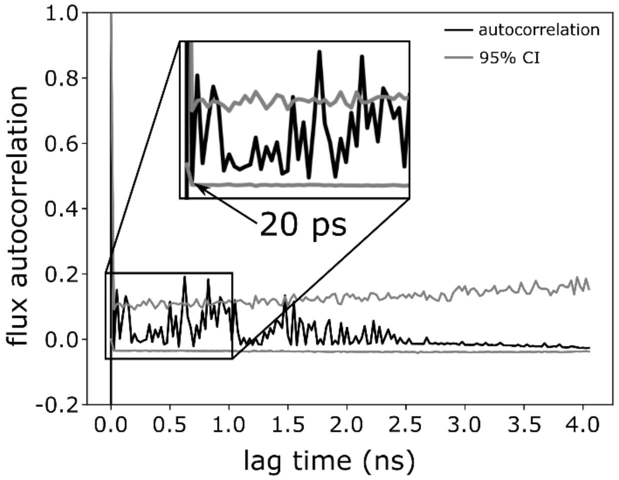

Fig. S3 Autocorrelation of the unbound-to-bound flux (black) with a 95% confidence interval (gray). The confidence interval was generated by calculating the autocorrelation of 1000 data sets (drawn with replacement from flux values in the latter half of the simulation) assuming each flux value is independent. The autocorrelation drops below the 95% confidence level at a lag time of τ = 20 ps (see inset), indicating that unbound-to-bound flux values are not significantly correlated in time in our simulation.

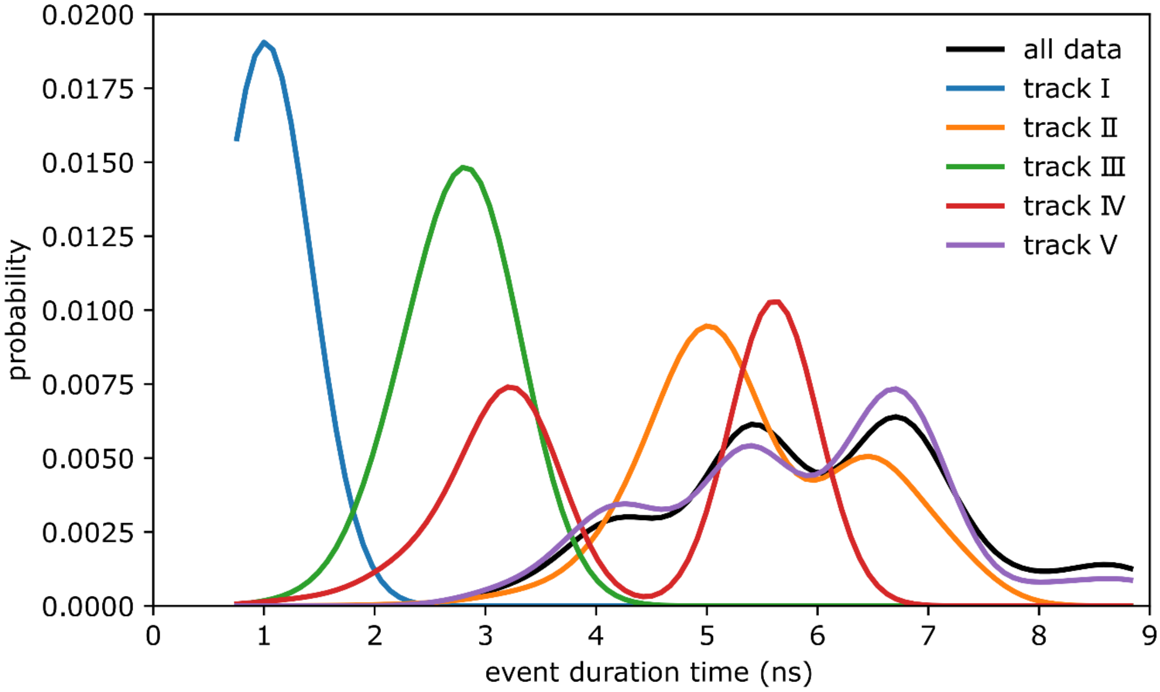

Fig. S4 Probability distributions of the event duration time for the barnase-barstar binding process from WE simulation. Distributions are shown for the entire simulation (black) as well as each of the five binding tracks (blue, orange, green, red, and purple). The probability distribution of each binding track is normalized according to the total weight of that distribution such that the sum over the probability distributions of all five binding tracks is unity.

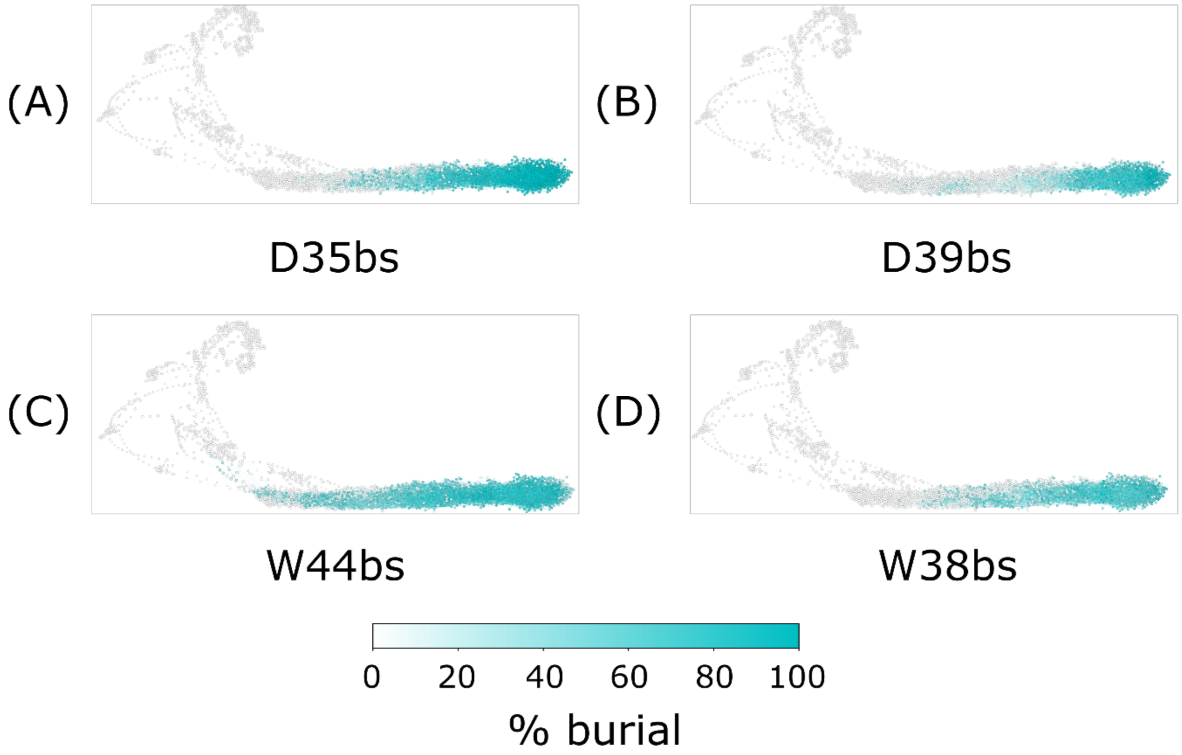

Fig. S5 Conformation space networks colored by the average percent burial of barstar (bs) residues upon binding barnase (bn) in each cluster. **(A)** D35bs, **(B)** D39bs, **(C)** W44bs, and **(D)** W38bs. The nodes of the networks are sized according to the total statistical weights of trajectories in each cluster. See **Fig. 5A** for locations of key states (unbound state, encounter complex, and bound state) in the networks.

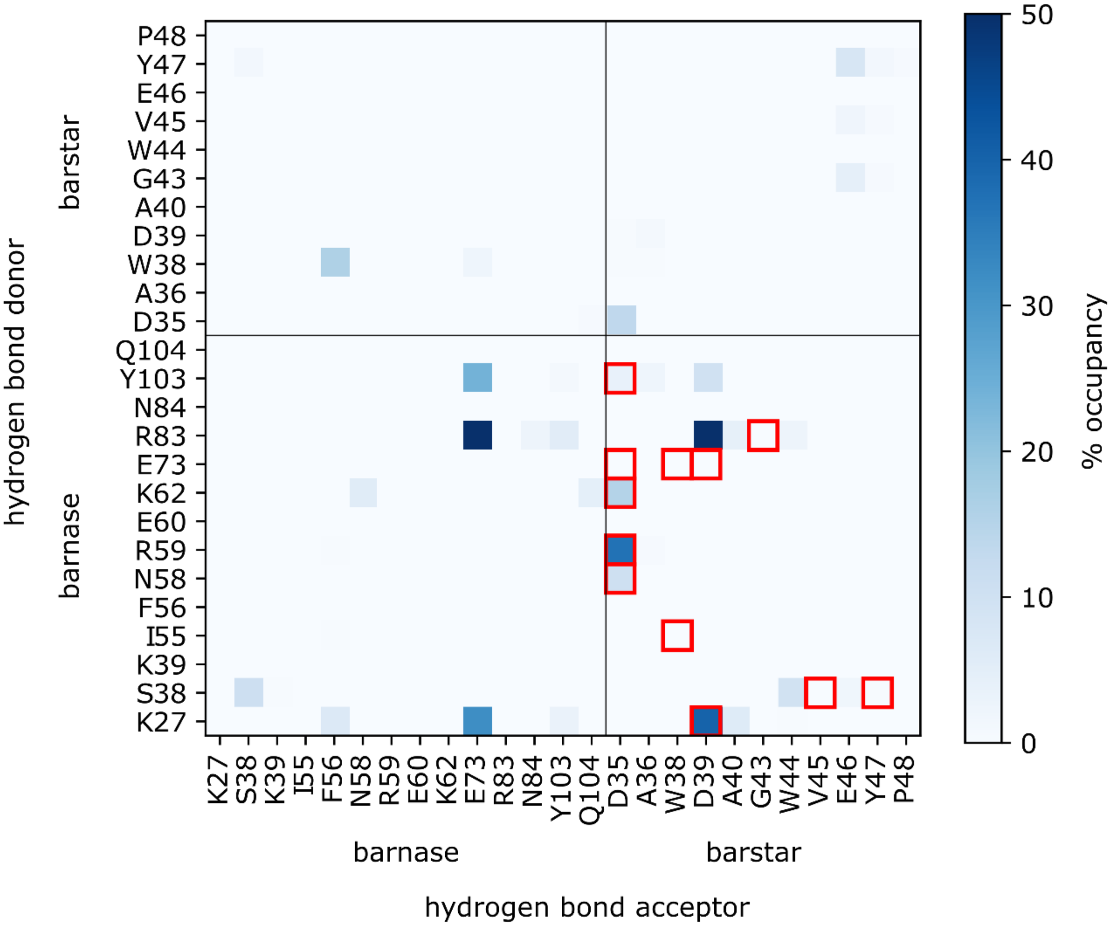

Fig. S6 Percent occupancy of solvent-mediated hydrogen bonds between barnase and barstar in the simulated bound-state ensemble. Hydrogen bonds that involve solvent at interfacial, crystallographic water positions are boxed in red.

Movie S1. Movie of a representative trajectory along the most probable binding track (track I) for barnase (blue) and barstar (orange) by WE simulation with conformations recorded every ps. This track is the most indirect, requiring the greatest extent of rotations of the two proteins in order to collide productively. Residues at the binding interfaces of barnase (S38bn and R59bn) and barstar (D35bs, D39bs, and W44bs) are highlighted in cyan and yellow, respectively. Also shown in the movie are surrounding water molecules with Na^+ and Cl^- ions to yield the experimental ionic strength of 50 mM NaCl.[1] Key features of the trajectory are highlighted in Fig. 4. The background music is “The Story Unfolds” by Jingle Punks and royalty-free. https://www.youtube.com/watch?v=0mhMwpLSUXY

References

1. G. Schreiber and A. R. Fersht, *Nat. Struct. Biol.*, 1996, **3**, 427-431.
